# Sex-specific differences in urinary incontinence associated factors in older adults: an analysis of the German health update study (GEDA 2019/2020-EHIS)

**DOI:** 10.1007/s00345-025-05999-2

**Published:** 2025-10-16

**Authors:** Leila Irik, Alexander Winter, Falk Hoffmann

**Affiliations:** 1 Department of Health Service Research, School of Medicine and Health Sciences, Carl von Ossietzky University Oldenburg, Oldenburg, Germany; 2 University Hospital for Urology, Klinikum Oldenburg, Department of Human Medicine, School of Medicine and Health Sciences, Carl von Ossietzky University Oldenburg, Oldenburg, Germany

**Keywords:** Urinary incontinence, Risk factors, Sex differences, Education, Physical impairment, Quality of life

## Abstract

**Purpose:**

Urinary incontinence (UI) is a common condition with significant health and societal implications, particularly among older adults. This study aims to analyze sex-specific differences in associated factors among adults aged 55+.

**Methods:**

Data from the cross-sectional German Health Update Study (GEDA 2019/2020-EHIS), conducted April 2019 to September 2020, were analyzed for participants aged 55 + years. Differences in UI prevalence by sex were analyzed along possible associated variables (e.g. BMI, education, depressive symptoms (PHQ-8 Score), chronic diseases (any condition expected to last for at least 6 months, self-reported), and limitations of activities of daily living, ADL). UI prevalence was analyzed with 95% confidence intervals (CI). To test for sex differences in associated factors, multivariable logistic regressions were performed.

**Results:**

Among 12,985 participants, UI prevalence was 14.7% and higher in females (17.8%, 95% CI 16.3–19.4) than males (11.1%, 95% CI 9.8–12.5). Female sex (OR 1.40, 95% CI 1.15–1.70, *p* < 0.001), age 85 + years (OR 7.07, 95% CI 4.88–10.24, *p* < 0.001), poor subjective health (OR 1.88, 95% CI 1.50–2.36, *p* < 0.001), ADL limitations (OR 1.75, 95% CI 1.24–2.48, *p* = 0.002), and depressive symptoms (OR 2.63, 95% CI 1.84–3.75, *p* < 0.001) were associated with UI in the multivariable regression. Sex-specific analysis found BMI ≥ 30 (OR 2.07, 95% CI 1.52–2.84, *p* < 0.001), lower education (OR 1.73, 95% CI 1.27–2.36, *p* < 0.001) and chronic diseases (OR 1.37, 95% CI 1.01–1.86, *p* = 0.042) associated with UI only in females.

**Conclusion:**

The self-reported UI prevalence in the general population is higher among females, but also every 9th male is affected. Due to its prevalence and possible impact on quality of life, attending physicians should have increased awareness of UI among older adults and actively offer treatment options.

## Introduction

Urinary incontinence (UI) is a common health condition affecting millions of people worldwide. Particularly prevalent in older adults, UI can lead to both medical and social problems [1, 2]. It can be described as a complex symptom rather than an isolated condition, that can develop into a disease in its own right when it causes complications and thus deteriorates the quality of life (QoL) of those affected [3, 4]. As a result of demographic changes, the prevalence of UI continues to increase [5], representing a growing societal burden in terms of economic costs and the demand for healthcare resources [6, 7]. In this context, several studies show a significant economic impact, that is expected to increase substantially in the coming decades as the population ages [1, 6].

The prevalence of UI is influenced by several factors, including demographic characteristics, lifestyle factors, mental health disorders or somatic diseases [8–10]. Previous studies have shown a significantly higher prevalence in females, which may be explained by sex-specific biological factors [11]. Conversely, UI in males is often associated with prostate disorders or surgical procedures such as radical prostatectomy (RP) [12, 13]. Despite these various causes, there is little literature on sex-specific differences in UI associated factors. Of the few studies that do make these comparisons, most focus on individual factors, like e.g. Body Mass Index (BMI) or Socioeconomic Status (SES) and rarely examine multiple determinants to identify which are most influential [14, 15]. However, the biopsychosocial implications of UI also play an important role in this context [9]. Factors such as limitations in activities of daily living (ADL) and mental disorders are an integral part in this relation [16, 17], although studies often lack a distinct examination of their association with UI. Overall, most studies have focused primarily on females or males following prostatectomy, overlooking the general population, and often lack direct sex-specific comparisons [13, 18]. This leaves a gap in understanding how UI prevalence is affected differently in males and females. In addition, much of the existing literature is already outdated [19, 20].

Therefore, the aim of this study was to investigate sex-specific differences in factors associated with UI among older adults in the general population in Germany.

## Methods

### Study design and data source

We used data from the cross-sectional German Health Update 2019/2020 (GEDA) study, conducted by the Robert Koch Institute (RKI), as part of the third wave of the European Health Interview Survey (EHIS) from April 2019 to September 2020 [21, 22]. The survey employed a structured, computer-assisted telephone interview method with dual-frame sampling of landline and cell numbers, targeting residents permanently based Germany, aged 15 and above. This mode of data collection was part of the standardized GEDA study design and allowed for broad population coverage and comparability with previous survey waves. A total of 23,001 interviews were completed (response 21.6%). Further details are described elsewhere [23]. For our analysis, we included participants aged 55 and older, as UI prevalence significantly increases from this age, and relevant questions were only available for this age group, resulting in a final sample of 12,985 observations.

### Questionnaire

The GEDA 2019/2020-EHIS questionnaire presented a comprehensive range of questions on health status, behavior and healthcare use, focusing on key areas such as chronic diseases, lifestyle and access to healthcare. The original questionnaire is available in the German-language article by Heidemann et al., published in the *Journal of Health Monitoring* (3/2021) [24].

UI was assessed by asking “Have you had urinary incontinence or problems controlling your bladder over the last 12 month?” (response options “yes” or “no”).

Health status was self-assessed, asking: “How is your health in general?”, which is also part of the internationally established SF-36 (Short Form Health Survey Instrument) [25, 26]. The response options ranged from “very good” to “very bad”. The responses “very good” and “good” are defined as a positively perceived subjective health [27], and were grouped as “rather good”, average or negative perceptions of health were grouped as “rather bad”. A chronic disease or long-term health problem was assessed using the question: “Do you have any chronic disease or a long-term health problem? This means diseases or health problems that have lasted for or are expected to last for at least 6 months” (response options “yes” or “no”).

Physical limitations were assessed using, Katz’s (1963) five Activities of Daily Living (ADL): (1) Eating or drinking, (2) Getting up from sitting on a bed or a chair, (3) Getting dressed and undressed, (4) Using the toilet, (5) Bathing or showering [28]. Difficulties were classified from “no difficulties” to “impossible”. Based on Gaertner et al., an ADL limitation was defined as an indication of at least major difficulties in at least one ADL [29].

To assess depressive symptoms, the internationally established 8-item Patient Health Questionnaire (PHQ-8) was utilized. Based on eight questions this instrument evaluates the frequency of symptoms over the last two weeks. Each question is scored from 0 (not at all) to 3 (nearly every day), resulting in a total score ranging from 0 to 24. A depression is generally assumed if the PHQ-8 Score is 10 or higher [30].

Body Mass Index (BMI, in kg/m^2^) was categorized according to WHO standards: underweight (< 18.5 kg/m^2^), normal body weight (18.5 to <25 kg/m^2^), overweight (25–30 kg/m^2^) and obesity (above 30 kg/m^2^), with underweight and normal body weight combined into ≤ 25 kg/m^2^ [31].

Socioeconomic data included biological sex, age groups (55–64, 65–74, 75–84, 85+), and education, based on the International Standard Classification of Education (ISCED), which classifies education into the following groups: low (up to 9 years of education), medium (up to 13 years of education) and high education (14 years or more) [32]. Education was chosen as an indicator of socioeconomic position, as it remains largely stable over the life course and is particularly suitable for use in older populations [33].

### Statistical analyses

The statistical analyses first described the baseline characteristics of the study population, stratified by sex. In a second step, UI prevalence was analyzed both overall and by sex, providing weighted percentages and 95% confidence intervals (CI). A multivariable logistic regression analysis was then conducted to identify associated factors of UI prevalence, using independent variables such as sex, age group, level of education, BMI, subjective health status, chronic disease or health problems, ADL limitations, and depressive symptoms (PHQ-8). Significant associations were determined by odds ratios, 95% CIs and p values < 0.05. Regressions were also run separately by sex. The test performance of the models was evaluated using the area under the receiver operating characteristic (ROC) curve (AUC).

According to the standard procedure defined by the GEDA 2019/2020-EHIS protocol, a weighting factor was applied to adjust the sample to reflect the German population. This factor considered age, sex, federal state, district type (as of December 31, 2019), and education, based on the 2017 Microcensus [23]. All analyses were performed with IBM SPSS Statistics, version 29.0 for Macintosh, using the “complex sampling” function.

The implementation of the GEDA 2019/2020-EHIS was approved by the ethics committee of the Charité Universitätsmedizin Berlin (application number EA2/070/19) [23].

## Results

### Characteristics of the study population

The study included 12,985 participants, 46.2% male (*n* = 5899) and 53.8% female (*n* = 7086). Most were aged 55–64 years and reported medium education (Table 1). The distribution of participants with normal weight and those with overweight was approximately equal (38.1% vs. 39.1%), while 22.7% reported a BMI ≥ 30. Overall, 56.3% rated their health as “rather good”, 61.3% reported a chronic illness or health problem in the previous 6 months. Only 4.9% reported ADL limitations. 18.5% of the participants reported mild depressive symptoms, only 6.9% reported moderate to severe depressive symptoms.

A sex-specific analysis showed females were generally older, had lower education levels, and reported more depressive symptoms than males. In contrast, males were more likely to be overweight. Other covariates showed only minor differences.

**Table 1:** Baseline characteristics, in % (weighted) with unweighted count

Category		Total		Male		Female	
		(*n* = 12,985)		(*n* = 5899; 46.2%)		(*n* = 7086; 53.8%)	
		%	*n*	%	*n*	%	*n*
Age group (years)	55–64	40.6	5128	43.7	2367	38.0	2761
(*n* = 12,985)	65–74	28.0	4308	28.6	2003	27.5	2305
	75–84	23.5	2966	22.0	1282	24.8	1684
	85+	7.8	583	5.6	247	9.7	336
Level of education^a^	Low	20.9	777	12.2	176	28.3	601
(*n* = 12,952)	Medium	55.7	5504	55.1	1908	56.1	3596
	High	23.4	6671	32.6	3798	15.5	2873
BMI (kg/m^2^)	< 25	38.1	5298	31.6	1952	43.9	3346
(*n* = 12,827)	25 to <30	39.1	5033	47.1	2755	32.2	2278
	≥ 30	22.7	2496	21.3	1167	24.0	1329
Subjective health	Rather good	56.3	8320	57.9	3867	54.9	4453
(*n* = 12,975)	Rather bad	43.7	4655	42.1	2027	45.1	2628
Chronic disease or health problem^b^	Yes	61.3	7761	60.5	3460	61.9	4301
(*n* = 12,935)	No	38.7	5174	39.5	2419	38.1	2755
ADL limitation^c^	Yes	4.9	420	3.8	153	5.9	267
(*n* = 12,984)	No	95.1	12,564	96.2	5745	94.1	6819
Depressive symptoms (PHQ-8)	No	74.6	9926	78.2	4777	71.4	5149
(*n* = 12,676)	Mild	18.5	2087	15.4	765	21.2	1322
	Moderate to severe	6.9	663	6.4	234	7.4	429

^a^Low (up to 9 years of education), medium (up to 13 years of education) and high education (14 years or more)

^b^Any chronic disease or a long-term health problem that has lasted/is expected to last for at least 6 months

^c^1. Eating/drinking, 2. Getting up from sitting on a bed/chair, 3. Getting dressed/undressed, 4. Using the toilet, 5. Bathing/showering

### Prevalence of urinary incontinence

Overall, 14.7% of participants reported UI (Table 2). UI prevalence rose with age (peaking at 36.5% in those aged 85+), higher BMI and lower education. Participants with “rather bad” subjective health, chronic diseases, ADL limitations, and depressive symptoms had significantly higher UI prevalences.

Stratified by sex, UI prevalence was higher in females than in males (17.8% vs. 11.1%). While increasing UI prevalence with poor subjective health status, chronic diseases, ADL limitations and depressive symptoms was found in both sexes, relevant differences were observed with education level and BMI. In females, UI prevalence increased with low education and higher BMI. For males, no statistically significant results were found for these variables.

**Table 2:** Prevalence of urinary incontinence by co-variable in total and by sex, in % (weighted) with 95% CI

Covariates		Prevalence of UI					
		Total (*n* = 12,964)		Male (*n* = 5887)		Female (*n* = 7077)	
		%	95% CI	%	95% CI	%	95% CI
Total		14.7	13.7–15.8	11.1	9.8–12.5	17.8	16.3–19.4
Age group (years)	55–64	6.8	5.7-8.0	5.4	4.0-7.3	8.1	6.7–9.7
(*n* = 12,964)	65–74	13.3	11.6–15.2	11.6	9.2–14.4	14.9	12.6–17.6
	75–84	22.7	20.4–25.3	17.6	14.6-21.21	26.6	23.3–30.2
	85+	36.5	30.7–42.8	27.0	19.8–35.5	41.2	33.5–49.4
Level of education^a)^	Low	23.9	20.4–27.8	15.7	10.3–23.3	26.9	22.7–31.5
(*n* = 12,932)	Medium	13.3	12.2–14.5	11.0	9.3–13.0	15.2	13.8–16.8
	High	9.6	8.8–10.5	9.4	8.3–10.6	10.0	8.8–11.3
BMI (kg/m^2^)	< 25	11.9	10.4–13.6	10.3	8.1–13.1	12.9	11.0-15.2
(*n* = 12,807)	25 to <30	14.0	12.5–15.7	10.5	8.7–12.6	18.5	16.0-21.4
	≥ 30	19.5	17.2–22.0	13.0	10.4–16.0	24.6	21.1–28.4
Subjective health	Rather good	8.1	7.1–9.3	5.9	4.8–7.2	10.2	8.6–12.0
(*n* = 12,954)	Rather bad	23.1	21.2–25.1	18.2	15.7–21.0	27.0	24.4–29.8
Chronic disease or health problem^b)^	Yes	18.2	16.8–19.7	14.1	12.3–16.2	21.6	19.6–23.7
(*n* = 12,915)	No	9.3	7.9–10.8	6.4	5.0-8.2	11.8	9.7–14.3
ADL limitation^c)^	Yes	44.0	37.0-51.1	33.7	23.7–45.3	49.7	40.8–58.6
(*n* = 12,963)	No	13.2	12.2–14.2	10.2	8.9–11.6	15.8	14.3–17.4
Depressive symptoms	No	10.7	9.7–11.8	8.0	6.8–9.4	13.2	11.7–15.0
(PHQ-8)	Mild	24.1	21.3–27.2	19.7	15.7–24.4	26.9	23.2–30.9
(*n* = 12,660)	Moderate to severe	29.4	24.1–35.3	27.1	19.0-37.1	31.1	24.6–38.4

^a^Low (up to 9 years of education), medium (up to 13 years of education) and high education (14 years or more)

^b^Any chronic disease or a long-term health problem that has lasted/is expected to last for at least 6 months

^c^1. Eating/drinking, 2. Getting up from sitting on a bed/chair, 3. Getting dressed/undressed, 4. Using the toilet, 5. Bathing/showering

### Associated factors of urinary incontinence

The multivariable logistic regression (Table 3) confirmed that female sex was associated with UI (OR 1.40, *p* < 0.001). The odds of having UI were significantly higher in those aged “85+” compared to “55–64 years” (OR 7.07, *p* < 0.001), and a “low” education level (OR 1.54, *p* = 0.002) compared to the reference group. Participants with “rather bad” subjective health had higher odds (OR 1.88, *p* < 0.001) of having UI, as did those with chronic diseases (OR 1.33, *p* = 0.017), ADL limitations (OR 1.75, *p* = 0.002), or “moderate to severe” depressive symptoms (OR 2.63, *p* < 0.001).

Sex-specific analysis showed that higher BMI (OR BMI 25 to <30: 1.57, *p* = 0.002; OR BMI ≥ 30: 2.07, *p* < 0.001; reference group BMI < 25), chronic diseases (OR 1.37, *p* = 0.042), and a low level of education (OR 1.73, *p* < 0.001) were significantly associated with UI only among females but not in males.

The test performance (AUC) was 0.776 (95% CI 0.763–0.788) in the total sample, 0.770 (95% CI 0.753–0.786) in females, and 0.777 (95% CI 0.757–0.796) in males, indicating satisfactory discrimination.

**Table 3:** Multivariable logistic regression for the probability of having urinary incontinence in total and divided by sex

Variable		Total			Male			Female		
		(*n* = 12,448)			(*n* = 5,714)			(*n* = 6,734)		
		OR	95% CI	*p* value^d^	OR	95% CI	*p* value^d^	OR	95% CI	*p* value^d^
Sex (ref. male)	Female	1.40	1.15–1.70	**< 0.001**						
Age group (years) (ref. 55–64)	65–74	2.31	1.78–2.98	**< 0.001**	2.48	1.61–3.83	**< 0.001**	2.17	1.59–2.95	**< 0.001**
	75–84	4.08	3.20–5.21	**< 0.001**	3.78	2.46–5.82	**< 0.001**	4.22	3.16–5.62	**< 0.001**
	85+	7.07	4.88–10.25	**< 0.001**	6.30	3.48–11.42	**< 0.001**	7.42	4.68–11.76	**< 0.001**
Level of education^a^ (ref. high level)	Medium	1.12	0.95–1.32	0.185	1.04	0.81–1.34	0.752	1.21	1.00-1.48	0.053
	Low	1.54	1.17–2.02	**0.002**	1.26	0.72–2.19	0.417	1.73	1.27–2.36	**< 0.001**
BMI (kg/m^2^) (ref. BMI < 25)	25 - <30	1.37	1.09–1.72	**0.008**	1.07	0.75–1.54	0.708	1.57	1.18–2.09	**0.002**
	≥ 30	1.69	1.31–2.19	**< 0.001**	1.17	0.76–1.79	0.479	2.07	1.52–2.84	**< 0.001**
Subjective health (ref. rather good)	Rather bad	1.88	1.50–2.36	**< 0.001**	2.13	1.44–3.17	**< 0.001**	1.70	1.29–2.23	**< 0.001**
Chronic disease or health problem^b^ (ref. no)	Yes	1.33	1.05–1.69	**0.017**	1.31	0.89–1.92	0.170	1.37	1.01–1.86	**0.042**
ADL limitation^c^ (ref. no)	Yes	1.75	1.24–2.48	**0.002**	1.90	1.12–3.23	**0.017**	1.71	1.10–2.67	**0.018**
Depressive symptoms (PHQ-8)	Mild	1.81	1.44–2.28	**< 0.001**	1.87	1.24–2.81	**0.003**	1.78	1.35–2.33	**< 0.001**
(ref. no)	Moderate to severe	2.63	1.84–3.75	**< 0.001**	3.06	1.72–5.44	**< 0.001**	2.37	1.53–3.69	**< 0.001**

^a^Low (up to 9 years of education), medium (up to 13 years of education) and high education (14 years or more)

^b^Any chronic disease or a long-term health problem that has lasted/is expected to last for at least 6 months

^c^1. Eating/drinking, 2. Getting up from sitting on a bed/chair, 3. Getting dressed/undressed, 4. Using the toilet, 5. Bathing/showering

^d^coefficientsdenote significance at the 5% level

## Discussion

In this population-based study, the self-reported prevalence of UI is higher among females (17.8%), but also every 9th male, aged 55 + years is affected (11.1%). Poor health, ADL limitations and depressive symptoms were associated with UI in both sexes. Among females, higher BMI and poor education were significantly associated with increased UI risk. These effects were not observed among males.

### Prevalence of UI

The overall prevalence of UI in our study was 14.7% and increased with age up to 36.5% in those aged 85 years and older. This increase is consistent with existing literature. A multinational study reported an increasing prevalence of UI with age in both females and males [34]. They also reported a higher prevalence of UI in females (19.3%) than in males (10.4%) over the age of 60 years, which is consistent with our findings (17.8% vs. 11.1%) [34]. However, few studies have investigated these sex differences in the general population. In females, UI is typically attributed to childbirth, age- or menopause-related changes in the pelvic floor, contributing to a higher prevalence [11], whereas in males, it is often associated with urological conditions such as prostatic hyperplasia (BPH), prostate cancer and its treatment [12, 13]. RP has often been described as a primary cause of UI [35]—the ProtecT study even found that 46% of males reported UI six months after RP, compared with 1% at baseline [36, 37]. However, studies report up to 18.8% prevalence of UI in males even before treatment, which shows that male UI is not solely due to iatrogenic causes [38]. Furthermore, in addition to BPH, age-related changes such as pelvic floor weakness or neurological diseases also contribute to the development of male UI [39]. This is illustrated, for example, by overactive bladder (OAB), where the prevalence in males approaches that in females in later life. Although OAB with incontinence remains more prevalent in females, these findings suggest that the etiology of male UI cannot be attributed solely to outlet obstruction or iatrogenic causes, but must also take age-related physiological changes into account [40]. Despite this fact, non-iatrogenic causes often play a subordinate role in research and clinical practice, where addressing UI in general is often neglected. Studies show that physicians frequently feel uncomfortable addressing UI and tend to avoid the topic in their patient-interactions [41].

### Sex-specific differences in associated factors of UI

The sex-specific logistic regression showed that higher BMI significantly increased the risk of UI in females, but not males. This aligns with existing literature, primarily focused on females, confirming the influence of weight on UI prevalence [42, 43]. Research in males, although often limited to UI as an outcome of RP, supports our findings. For example, Brown et al. found that BMI had no significant effect on UI after RP [44]. Similar results have been observed in other studies [45]. Nevertheless, only few studies have made a sex-specific comparison within the general population; those that have are largely consistent with our findings. A cross-sectional study found that higher BMI increased the odds of UI in females but not in males [14], suggesting that increased abdominal pressure on the pelvic floor may be the reason. Others support this notion, stating that a stronger pelvic floor fixation mitigates these effects in males [46]. Beyond BMI, previous research has demonstrated a clear association between metabolic syndrome (MetS) and UI subtypes, with stronger and broader associations in females [47]. However, comparisons across studies remain difficult, due to the use of different UI outcome variables (ranging from overall UI to distinct subtypes) and the heterogeneity in obesity-related metrics such as BMI, waist circumference, or waist-to-hip ratio.

Regarding the level of education, our study found that lower levels of education increased UI prevalence only in females. This is consistent with existing research, where the relationship between low education and higher UI prevalence among females is documented, partly due to a lack of awareness about treatable health conditions and reduced healthcare utilization [48]. Others support this notion, showing that lower education correlates with reduced healthcare use and increased incontinence rates among females [49]. Conversely education appears to have less of an influence on male UI, as it is less amenable to health education and lifestyle changes, due to common underlying causes [13]. Furthermore, studies show that pelvic floor muscle training before prostatectomy accelerates short-term continence, but does not improve long-term continence rates, supporting this assumption [50].

Overall, our findings highlight significant differences in the factors associated with UI between the sexes, that should be considered when assessing this condition. While particular attention should be paid to females with low education and obesity, the increasing prevalence of UI in males and its limited modifiability with conservative measures and lifestyle changes should not be overlooked.

### Biopsychosocial factors

Our study identified ADL limitations and depression as biopsychosocial factors significantly associated with UI in both sexes. While individual ADL such as “using the toilet” are clearly linked to UI, our analysis of general ADL limitations—defined as significant difficulty in at least one ADL [29]—confirms overall physical impairments are significantly associated with UI. This aligns with findings from Schumpf et al. (2017), who showed ADL impairments were more strongly associated with UI than cognitive impairments or other comorbidities [51]. Similarly, other studies found females with reduced physical performance more likely to report weekly incontinence [52].

Additionally, depression as measured by the PHQ-8 was associated with increased UI risk for both sexes. The connection between depression and UI appears to be bidirectional. While some studies highlight the impact of UI on depression [53], others propose that these conditions share a common neurochemical pathogenesis, potentially explaining the significant influence of depression on UI found in our study [54]. Steers et al. postulate that neurotransmitters such as serotonin, associated with mood and emotion, may also affect bladder function. Thus, changes or imbalances in these neurotransmitters due to depression could simultaneously cause bladder overactivity, potentially leading to urge incontinence [54]. However, comparing study results remains challenging, due to different instruments used to measure depression or mental health. Nevertheless, our study provides important insights into these co-occurring conditions and underscores the strong link between physical and psychological conditions. Their interdependence and mutual influence underscore the importance of a holistic, biopsychosocial approach to UI.

### Strengths and limitations

Using data from the GEDA 2019/2020-EHIS study, we examined a large, representative sample of both sexes from the general population in Germany. Using standardized methods and a comprehensive data set, our study offers valuable insight into sex-specific differences in UI associated factors. Despite these insights, there are some limitations. First, UI was self-reported, based on a simple yes/no-question, asking about “involuntary loss of urine or problems controlling your bladder” over the past 12 months. This broad wording may not clearly distinguish between UI and other lower urinary tract symptoms (LUTS), e.g. urgency or frequency. Furthermore, it does not capture information on severity, frequency, or subtype (e.g. stress, urge). This may have resulted in misclassification by participants, potentially affecting prevalence estimates and group comparisons. Furthermore, data on sex-specific characteristics—such as gravidity or parity in females or prostate diseases in males—where not available. In addition, limited information on prior surgery, treatment history, and on medications used complicate interpretation. Additionally, the dataset did not include information on ethnicity or racial identity, which may influence UI prevalence. Although country of birth was available, it does not adequately capture ethnic background and was therefore not included in our analysis. Furthermore, interviews were conducted via telephone, which might have excluded people with high care needs or cognitive impairment, underrepresenting these individuals. Social or cultural factors may also affect responses. The topic’s sensitivity could lead to underreporting, as some people may hesitate to discuss their incontinence. However, this effect may be less pronounced on the phone due to greater anonymity.

## Conclusion

UI is a relevant health issue among older adults in Germany, affecting approximately one in six females and one in nine males. Our results highlight important sex-specific associations, with BMI, education, and chronic disease being significant only in females. Psychosocial and functional factors such as depressive symptoms, self-rated health, and ADL limitations were relevant in both sexes. These findings underline the importance of proactively addressing UI in clinical care, particularly in underdiagnosed groups such as males, where incontinence is often viewed primarily as iatrogenic. Further research should explore sex-specific barriers to care and health-seeking behavior to improve targeted prevention and intervention—especially since most existing studies focus exclusively on females.

## Data Availability

The data supporting the results presented in this manuscript were obtained from the German Health Update (GEDA) 2019/2020 study conducted by the Robert Koch Institute (RKI). The dataset is available as a Scientific Use File (SUF) and can be requested from the Research Data Center (FDZ) of the RKI at the following URL: https://doi.org/10.7797/31-201920-1-1-1.
